# CIS2/377: INTRAMED: An Integrated Environment System for a Medical Course

**DOI:** 10.2196/jmir.1.suppl1.e7

**Published:** 1999-09-19

**Authors:** M deA Novaes, PHE Silva, PGAP Galvão, SJ Medeiros, F Pedrosa

**Affiliations:** ^1^Universidade Federal de Pernambuco (UFPE), RecifeBrasil

**Keywords:** Intranet, Medical Education, Problem-Based Learning, Provider of Information

## Abstract

**Introduction:**

Education in medicine is being revolutionized by recent achievements in information technology. New research findings for prevention, diagnosis and treatment of diseases are increasing considerably the amount of information required in by medical courses. The use of information technologies has modified access and integration of this diversity of information minimizing the overload of the medical curriculum.

**Goals:**

Our goal is to develop a web-based environment, INTRAMED, to address the new teaching and learning procedures in the medical school of the Federal University of Pernambuco (UFPE).

**Methodology and Equipment:**

The project will provide a new way for the medical students to deal with the excess of information using recent information technologies in the health field both as an educational and informative tool. This includes the development of individual tools (integrating complex objects such as audio, fix images, video, and Internet access) for each discipline in the medical course. These tools will be stored in an SQL database server using a Microsoft NT environment. Each student or teacher will have their own personal login and password to access their data. The Internet access will be offered through the medical course homepage. This project involves the mutual co-operation of the Technology, Information and health group (TIS) and the teachers responsible for the disciplines. It will be developed at the LIKA laboratories. The system will be available for the users on the Computer Laboratory of the Medical Course composed by 60 microcomputers connected to the Internet.

**Results:**

Provide access to information on all disciplines included in the course, students' scores, activities schedule, a fast way of communicating to teachers, students and course co-ordinators. The use of a Web environment to support teaching and learning activities will provide the necessary speed and flexibility to accomplish the next step in supporting our medical school activities.

